# Supplementary material for: Co-designed web platform (TeenFit) to identify physical activity mobile apps for Spanish adolescents: a study protocol
Source: BMJ Open. 2026 Apr 28;16(4):e114465. doi: 10.1136/bmjopen-2025-114465 (PMC13141059; doi:10.1136/bmjopen-2025-114465)
Supplement: online supplemental file 2 [file bmjopen-16-4-s002.docx]

**Supplemental Material 2**

**Adaptation of the acceptability questionnaire:**

1. **What do you think about the title of the website?**
   (Open-ended question)
2. **The language used on the website was easy to understand.**

- Strongly disagree
- Disagree

Neither agree nor disagree

Agree

- Strongly Agree

1. **It was easy to navigate through the website and to choose the app I was looking for.**

- Strongly disagree
- Disagree

Neither agree nor disagree

- Agree
- Strongly Agree

1. **The information presented was organized coherently and clearly.**

- Strongly disagree
- Disagree

Neither agree nor disagree

- Agree
- Strongly agree

1. **The overall quality of the website design and its content seemed to me:**

- Strongly disagree
- Disagree

Neither agree nor disagree

- Agree
- Strongly agree

1. **The website objectives were clear.**

- Strongly disagree
- Disagree

Neither agree nor disagree

- Agree
- Strongly agree

1. **The website content was consistent with the stated objectives and with the intended audience.**

- Strongly disagree
- Disagree

Neither agree nor disagree

- Agree
- Strongly agree

1. **I found the website interesting.**

- Strongly disagree
- Disagree

Neither agree nor disagree

- Agree
- Strongly agree

1. **This website met my expectations.**

- Strongly disagree
- Disagree

Neither agree nor disagree

- Agree
- Strongly agree

1. **The description of each app and its features was clear and easy to understand, avoiding possible misinterpretations.**

- Strongly disagree
- Disagree

Neither agree nor disagree

- Agree
- Strongly agree

1. **The titles, buttons, and links on the website clearly matched the content or pages they led to.**

- Strongly disagree
- Disagree
- Undecided

Neither agree nor disagree

Agree

- Strongly agree

1. **The information and recommendations on the website were useful for helping me understand how to choose and use physical activity apps.**

- Strongly disagree
- Disagree

Neither agree nor disagree

- Agree
- Strongly agree

1. **The tools or features provided (e.g., filters, app descriptions, ratings) helped me evaluate which apps were most suitable for me.**

- Strongly disagree
- Disagree

Neither agree nor disagree

- Agree
- Strongly agree

1. **Do you think this website has helped you improve your ability to practice more physical activity by learning how to find relevant and reliable physical activity apps in the marketplace?**

- Strongly disagree
- Disagree

Neither agree nor disagree

- Agree
- Strongly agree

1. **I would recommend this website to other people.**

- Strongly disagree
- Disagree

Neither agree nor disagree

- Agree
- Strongly agree

1. **The amount of time required to find the physical activity app that suits you was:**

- Appropriate
- Excessive, required too much time
- Insufficient, required too little time

1. **Please provide a summary of the website’s strengths and weaknesses.**
   (Open-ended question)
2. **Please provide brief suggestions on how to improve the website.**
   (Open-ended question)
3. **What are the main points you have learned throughout this website?**
   (Open-ended question)
