# Supplementary material for: Co-designed web platform (TeenFit) to identify physical activity mobile apps for Spanish adolescents: a study protocol
Source: BMJ Open. 2026 Apr 28;16(4):e114465. doi: 10.1136/bmjopen-2025-114465 (PMC13141059; doi:10.1136/bmjopen-2025-114465)
Supplement: Supplementary file 3 [file bmjopen-2025-114465.reviewer_comments.pdf]

PEER REVIEW HISTORY

BMJ Open publishes all reviews undertaken for accepted manuscripts. Reviewers are asked to complete a checklist review form and are provided with free text boxes to elaborate on their assessment. These free text comments are reproduced below.

ARTICLE DETAILS

Title (Provisional)

A co-designed web platform (TeenFit) to identify physical activity mobile apps for Spanish adolescents: a study protocol

Authors

Seitero, Anna; Ruiz Moreno, Alicia; Alcarazo, Adriana; Garrido Gonzalez, Carmen; Ballesta-Rodríguez, María I.; Henriksson, Pontus; Lima, Rodrigo Antunes; Álvarez-Pérez, Yolanda; Reigal, Rafael E.; Campos-Paino, Henar; Bellón, Juan A.; Motrico, Emma; Flores-López, María; Rodriguez-Ayllon, María

VERSION 1 - REVIEW

**Reviewer**	**1**
**Name**	**Pedras, Susana**
**Affiliation**	**Universidade Lusíada Norte, Instituto de Psicologia e Ciências da Educação**
**Date**	**31-Dec-2025**
**COI**	**None**

For the authors,

I would like to express my sincere appreciation for your thorough and well-developed protocol on the development and implementation of the TeenFit website. Your work addresses a critical gap in promoting adolescent health, especially at a time when sedentary lifestyles remain a widespread challenge and digital solutions proliferate without proper scientific validation.

The decision to focus on fitness apps, an area lacking validated repositories comparable to those available in mental health, highlights both the relevance and the timeliness of the Protocol.

I was particularly pleased with the participatory design approach adopted. Involving teenagers along with caregivers, educators, and healthcare professionals ensures that the TeenFit website is not only evidence-based but also meaningful, usable, and responsive to the real needs of its users. The workshop structure, the integration of qualitative and

quantitative data, and the attention to the development and implementation phases reflect a rigorous and comprehensive methodology.

The plan to incorporate findings from ongoing systematic reviews further strengthens the scientific basis of the TeenFit platform. Similarly, the use of reflective thematic analysis to explore the barriers and facilitators of implementation demonstrates a thoughtful commitment to understanding the complexities of real-world adoption.

Overall, this study represents an important and much-needed contribution to the field of adolescent health promotion and digital health. I look forward to following the progress of the TeenFit website and the valuable insights your work will undoubtedly generate for research and practice.

Best regards,

**Reviewer**	**2**
**Name**	**Hesketh, Katie**
**Affiliation**	**Liverpool John Moores University**
**Date**	**20-Jan-2026**
**COI**	**None**

Minor comments

1) Clarification of app selection and governance

In the Introduction, the manuscript refers to physical activity apps being "pre-screened by an expert committee" (page 7–8, lines ~158–165; page 8, lines ~188–192).

It would be helpful to briefly clarify the composition of this expert committee (e.g. disciplinary backgrounds) and whether the quality criteria used for app inclusion (e.g. evidence base, safety, data privacy) will be visible to end users on the TeenFit website.

This could be addressed with one or two additional sentences in the Introduction or Aims section.

2) Long-term sustainability and updating of the TeenFit website

In the Discussion (page 22, lines ~445–455), the authors may wish to briefly acknowledge the dynamic nature of the mobile app marketplace.

A short reflection on how the TeenFit platform or app catalogue might be updated or governed in the future (beyond the scope of the current study) would strengthen the manuscript and demonstrate awareness of sustainability considerations.

3) Generalisability beyond the Spanish context

The limitations section of the Discussion (page 22, lines ~468–478) clearly acknowledges the use of convenience sampling.

The authors may consider adding a brief statement noting that while the app catalogue focuses on the Spanish market, the co-design methodology and implementation framework may be transferable to other cultural or national contexts.

4) Streamlining between Aims and Objectives

There is some repetition between the Aims and Objectives sections (page 8–9, lines ~175–205).

5) Minor editorial consistencies

There are a few minor typographical and formatting inconsistencies (e.g. hyphenation, spacing artefacts such as "real world") throughout the manuscript, particularly in the Methods and References sections.

VERSION 1 - AUTHOR RESPONSE

Reviewer: 1

For the authors,
I would like to express my sincere appreciation for your thorough and well-developed protocol on the development and implementation of the TeenFit website. Your work addresses a critical gap in promoting adolescent health, especially at a time when sedentary lifestyles remain a widespread challenge and digital solutions proliferate without proper scientific validation.
The decision to focus on fitness apps, an area lacking validated repositories comparable to those available in mental health, highlights both the relevance and the timeliness of the Protocol.
I was particularly pleased with the participatory design approach adopted. Involving teenagers along with caregivers, educators, and healthcare professionals ensures that the TeenFit website is not only evidence-based but also meaningful, usable, and responsive to the real needs of its users. The workshop structure, the integration of qualitative and quantitative data, and the attention to the development and implementation phases reflect a rigorous and comprehensive methodology.
The plan to incorporate findings from ongoing systematic reviews further strengthens the scientific basis of the TeenFit platform. Similarly, the use of reflective thematic analysis to explore the barriers and facilitators of implementation demonstrates a thoughtful commitment to understanding the complexities of real-world adoption.
Overall, this study represents an important and much-needed contribution to the field of adolescent health promotion and digital health. I look forward to following

the progress of the TeenFit website and the valuable insights your work will undoubtedly generate for research and practice.

Authors' reply: Thank you. We very much appreciate your comments regarding the value of this study and its rigorous design.

Reviewer: 2

1) Clarification of app selection and governance
In the Introduction, the manuscript refers to physical activity apps being "pre-screened by an expert committee" (page 7–8, lines ~158–165; page 8, lines ~188–192).

It would be helpful to briefly clarify the composition of this expert committee (e.g. disciplinary backgrounds) and whether the quality criteria used for app inclusion (e.g. evidence base, safety, data privacy) will be visible to end users on the TeenFit website.

This could be addressed with one or two additional sentences in the Introduction or Aims section.

Authors' reply: Thank you for your important comment. We agree that providing information about the review committee would help increase the transparency of the process. Consequently, we have added details describing the professionals (psychology, sports medicine, medical doctor, and nurse) among the group of app reviewers [page 6, lines 120–122]. We have also clarified in the manuscript [pages 18-19, lines 419–420] that the results of the quality review of each app will be visible to users of the TeenFit website.

Page 6, lines 120–122: The reviewer team covers different professional areas, including three psychologists, four sports scientists, two nurses, and one individual who is both a sport scientist and a psychologist.

Pages 18-19, lines 419–420: The results of the quality review for each app will be provided on the TeenFit website.

2) Long-term sustainability and updating of the TeenFit website
In the Discussion (page 22, lines ~445–455), the authors may wish to briefly acknowledge the dynamic nature of the mobile app marketplace.

A short reflection on how the TeenFit platform or app catalogue might be updated or governed in the future (beyond the scope of the current study) would strengthen the manuscript and demonstrate awareness of sustainability considerations.

Authors' reply: Again, thank you very much for your insightful comment. While the website allows us to continuously add new apps and remove those that are no longer

available on the market, the results of this and future studies on the TeenFit website will guide our next steps to support scalable implementation and long-term operation. We have expanded the Discussion [page 19, lines 431–435] to outline these aspects.

> Page 19, lines 431-435: Overall, TeenFit will produce a validated catalog of physical activity apps, a user-centered web tool, and insights to support its implementation. While the website provides an opportunity to add and remove apps to ensure accuracy, future studies on the effects on physical activity of the TeenFit website are needed to determine whether and how a scalable and sustainable implementation can be supported.

3) Generalisability beyond the Spanish context
The limitations section of the Discussion (page 22, lines ~468–478) clearly acknowledges the use of convenience sampling.

The authors may consider adding a brief statement noting that while the app catalogue focuses on the Spanish market, the co-design methodology and implementation framework may be transferable to other cultural or national contexts.

Authors' reply: Thank you for highlighting the knowledge contribution our project can offer to other interventionists pursuing similar initiatives. As suggested, we have added a brief statement [page 19, lines 421–430] regarding the limitation of including apps that focus solely on the Spanish market, as well as the potential to gain valuable insights that are transferable to other contexts.

> Page 19, lines 421-430: The focus on the Spanish market also limits the value of the TeenFit website beyond Spain. Although effort will be made to enhance diversity, the study includes only Spanish-speaking individuals and relies on a convenience sampling strategy, with recruitment restricted to a limited geographic area. This may not fully represent populations in rural areas or reflect broader sociocultural diversity, which is a limitation. Nevertheless, we believe that the sample size is sufficient for co-design and exploratory research, allowing meaningful input for website development. Furthermore, the methodological insights gained from this project regarding the co-design and implementation of a website to aid in the identification of evidence-based mobile apps may be valuable for similar initiatives, as such examples are still limited.

4) Streamlining between Aims and Objectives
There is some repetition between the Aims and Objectives sections (page 8–9, lines ~175–205).

Authors' reply: Thank you. We agree and have revised these sections by merging the text and removing the subheading "Objectives" [pages 6-7, lines 139-146].

> Page 6-7, lines 139-146: The overall aim of this project is to develop a co-designed website, in collaboration with adolescents, caregivers, educators, and health professionals, aimed at supporting young people to promote physical activity using third-party mobile applications that have been pre-screened by an expert committee to ensure their quality. The specific aims of the project are to:
>
> 1. Co-design a website, named the TeenFit website, that facilitates the selection of physical activity apps tailored to adolescents' needs.
> 2. Investigate the barriers and facilitators for the implementation of the TeenFit website in real-world settings (e.g., high schools, sports clubs, and healthcare settings).

5) Minor editorial consistencies

There are a few minor typographical and formatting inconsistencies (e.g. hyphenation, spacing artefacts such as "realworld") throughout the manuscript, particularly in the Methods and References sections.

Authors' reply: We appreciate your feedback. We have now corrected all identified inconsistencies.
